# The Prognostic Value of Lymph Node Downstaging Following Neoadjuvant Chemoimmunotherapy for Non-Small Cell Lung Cancer

**DOI:** 10.7150/jca.119881

**Published:** 2025-10-20

**Authors:** Marissa Guo, Mahmoud Abdel-Rasoul, Jeremy Chang, Aaron Guo, Ioana Baiu, Desmond M. D'Souza, Robert E. Merritt, Peter J. Kneuertz

**Affiliations:** 1 Division of Thoracic Surgery, Department of Surgery, The Ohio State University Wexner Medical Center, Columbus, OH 43210, USA.; 2 The Ohio State University Comprehensive Cancer Center, James and Solove Research Institute, Columbus, OH 43210, USA.; 3 Center for Biostatistics, The Ohio State University College of Medicine, Department of Biomedical Informatics, Columbus, OH 43210, USA.; 4 The Ohio State University College of Medicine, Columbus, OH, 43210, USA.

**Keywords:** non-small cell lung cancer, neoadjuvant chemoimmunotherapy, lymph node downstaging

## Abstract

**Background**: The use of immune checkpoint inhibitors (ICI) and other targeted molecular agents for non-small cell lung cancer (NSCLC) has led to unprecedented rates of major pathologic response and improvements in overall survival. The aim of this study was to evaluate the prognostic significance of lymph node downstaging following neoadjuvant chemoimmunotherapy for resectable NSCLC.

**Methods**: This study used retrospective data from the National Cancer Database (NCDB), which was queried for all patients diagnosed with NSCLC between 2017-2021 who underwent lung cancer surgery after receiving neoadjuvant chemoimmunotherapy. Only those staged as cN1 or cN2 were included. Patients were stratified according to post-therapy pathologic lymph node status, whether positive (ypN+) or negative (ypN-). Five-year overall survival (OS) was examined using Kaplan-Meier analyses with log-rank tests. Univariate and multivariate Cox regression analyses were conducted to identify significant predictors of survival.

**Results**: Of the total 621 patients, 229 (37%) were diagnosed with cN1 disease and 392 (63%) with cN2. With neoadjuvant chemoimmunotherapy, 59% of cN1 and 40% of cN2 patients were down-staged to ypN0. While 5-year OS was not significantly different according to clinical N stage (76% for cN1 vs. 63% for cN2, p=0.08), higher post-therapy nodal staging correlated with poorer long-term survival (5-year OS of 84% for ypN0, 64% for ypN1, and 51% for ypN2, p <0.001). On multivariate analysis, cN1 to ypN+ (HR 2.56, p=0.009) and cN2 to ypN+ (HR 3.09, p=0.001) were predictors of worse OS compared to cN1 to ypN-, while the difference was not statistically significant for cN2 to ypN- (HR 1.01, p=0.051). Among ypN- patients, similar 5-year OS was seen among those who received adjuvant chemoimmunotherapy and those who did not (82.2% vs. 86.2%, p = 0.26).

**Conclusion**: Patients receiving neoadjuvant chemoimmunotherapy for resectable NSCLC experience high rates of nodal down-staging. Achieving ypN0 status post-therapy strongly predicts favorable long-term survival in this population, while pretreatment cN stage becomes less prognostically relevant.

## Introduction

For patients with non-small cell lung cancer (NSCLC), surgical resection remains a cornerstone of curative intent therapy. Nevertheless, approximately 20% of patients with stage I-II NSCLC and over 50% of those with stage III NSCLC ultimately experience recurrences due to the presence of micrometastatic disease, highlighting the importance of systemic treatment in select cases 1. Within the past decade, the development of immune checkpoint inhibitors (ICI) and other targeted molecular agents has transformed the therapeutic landscape for NSCLC. The majority of NSCLC cases involving tumors that lack targetable driver mutations, immunotherapy using specific antibodies against programmed cell death protein 1 (PD-1) and its ligand (PD-L1) has been shown to be highly effective, becoming a key first-line treatment option 5-7. The combination of chemoimmunotherapy has been associated with major pathologic response, defined as ≤ 10% residual tumor tissue, in nearly 60% of patients 8. Multiple phase III trials have demonstrated improved event-free survival among patients receiving ICI immunotherapy in addition to chemotherapy as a part of neoadjuvant or perioperative treatment protocols 9-14.

The presence of lymph node metastases, which represents an integral component of TNM staging, has been recognized as a major predictor of long-term survival. Previous studies have demonstrated significant associations between nodal downstaging following neoadjuvant chemotherapy and subsequent improvements in overall, event-free, and disease-free survival 19-21. However, there have been limited investigations to date into the prognostic significance of nodal response following neoadjuvant chemoimmunotherapy, which is currently recommended for patients with stage II-III NSCLC. The rate of lymph node downstaging has been reported to be significantly higher following ICI therapy as compared to chemotherapy alone 9-18. Nodal response and post-therapy lymph node staging could help stratify patients and guide treatment decisions regarding adjuvant therapy. However, questions persist on whether patients should be treated differently according to their initial staging even after experiencing nodal downstaging with preoperative therapies. Therefore, we sought to evaluate the prognostic value of lymph node downstaging among patients with NSCLC who received neoadjuvant chemoimmunotherapy prior to surgical resection.

## Methods

**Figure 1:**
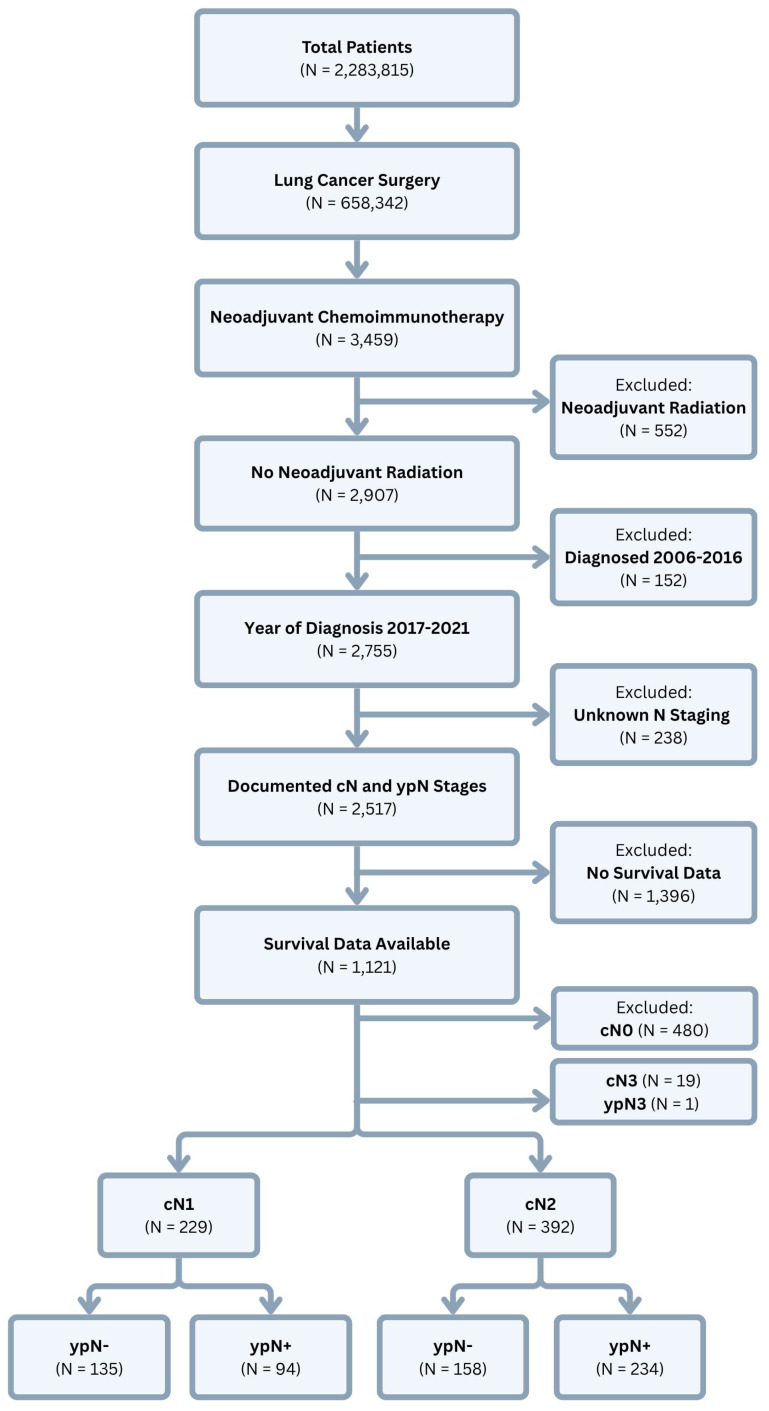
Study population selection from the National Cancer Database.

*Data Source and Study Population*. This study used retrospective data from the National Cancer Database (NCDB), which includes clinical and oncologic data from > 1,500 Commission on Cancer-accredited facilities across the United States. Clinical staging in the NCDB is classified using the American Joint Commission on Cancer (AJCC) 7^th^ edition for 2017 and the AJCC 8^th^ edition for 2018-2021. The NCDB was queried for all patients who were diagnosed with NSCLC between the years 2017-2021 and underwent lung cancer surgery after receiving neoadjuvant chemoimmunotherapy. Patients were excluded if they received neoadjuvant radiation or if they were documented to have clinical or pathologic N3 disease. Patients were also excluded if they had clinically negative nodes at the time of diagnosis (**Figure 1**).

**Figure 2:**
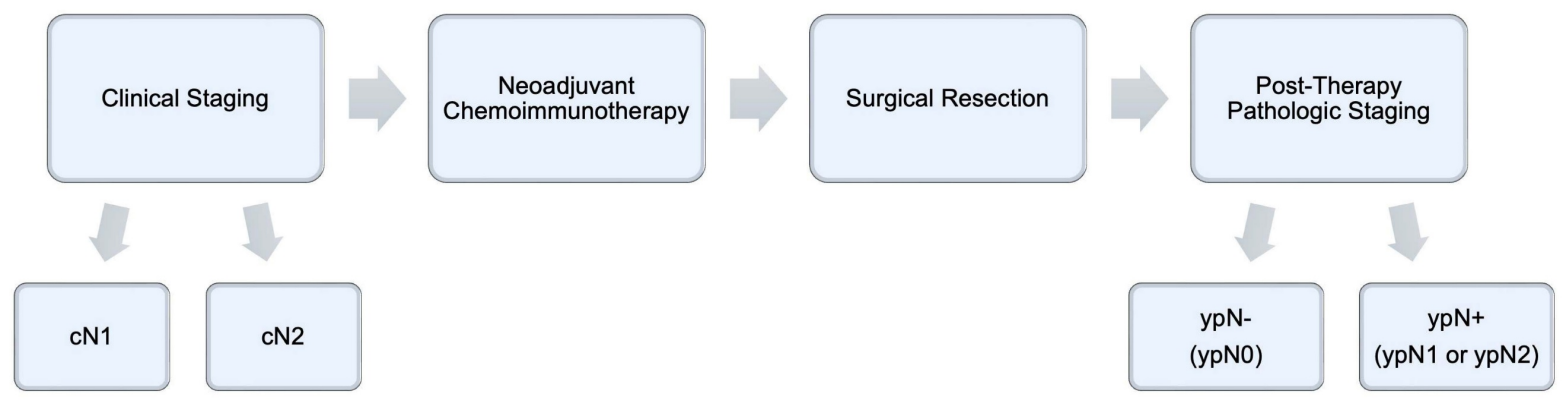
Temporal relationship between clinical staging, neoadjuvant therapy, surgical resection, and post-therapy pathologic staging.

*Study Variable Selection and Outcomes*. Patient demographic and clinical characteristics, including age at the time of diagnosis, sex, race, facility type, insurance status, and Charlson-Deyo comorbidity index score, were obtained from the database. Extracted oncologic variables included tumor histology, tumor size, clinical T and N stages, post-therapy pathologic T and N stages, and details relating to cancer treatment, such as the time to initiation of systemic therapy and immunotherapy; time to surgical resection; type of operation (lobectomy or bilobectomy, wedge resection or segmentectomy, and pneumonectomy); surgical approach (open thoracotomy, robotic-assisted thoracoscopic surgery, or video-assisted thoracoscopic surgery); length of hospital stay after surgery; 30- and 90-day postoperative mortality; surgical margin status; number of regional nodes examined and returned positive; and whether or not patients received adjuvant chemoimmunotherapy and/or radiation. Patients were grouped according to clinical lymph node staging (cN1 versus cN2), then further stratified by their post-therapy pathologic lymph node status, whether positive (ypN1 and ypN2) or negative (ypN0) (**Figure 2**). The primary endpoint examined was overall survival (OS) after diagnosis. Survival data were censored at a follow-up of 5 years.

*Statistical Analysis*. Descriptive statistics are presented as frequencies and percentages for categorical variables and medians with interquartile range (IQR) for non-normally distributed continuous data. Fisher's exact test was used for comparisons of categorical variables, while comparisons of continuous data were made using the Wilcoxon rank-sum test. Kaplan-Meier curves with log-rank tests were performed to examine for differences in OS based on clinical and post-therapy pathologic N stage. To investigate the effect of nodal downstaging on OS, separate Kaplan-Meier analyses were performed for patients with cN1 and cN2 disease with further stratification according to post-therapy nodal status. Pairwise comparisons of survival distributions were performed using the log-rank test, and p-values were adjusted using the Holm-Bonferroni method to correct for multiple testing. Univariate and multivariate Cox regression were used to identify factors associated with OS. The proportional hazards assumption was confirmed by examination of individual covariate and global Schoenfeld residuals to confirm a non-significant relationship with time. Results are reported as hazard ratios (HR) with 95% confidence intervals (CI). A two-tailed p < 0.05 was considered statistically significant. All statistical analyses were performed using R 4.2.2 software (R Foundation for Statistical Computing; packages: *gtsummary*, *survival*, *survminer*, *coxphf*).

## Results

The final analysis included 621 patients. Of these, 229 (37%) were diagnosed with cN1 disease and 392 (63%) were staged as cN2. Patient demographic and clinical characteristics are described in full in **Tables 1A and 1B**. Among cN1 patients, those with positive nodes post-therapy (ypN+) were more likely to be male, undergo surgical resection sooner, undergo pneumonectomy, have positive surgical margins and higher post-therapy pathologic T staging, and receive adjuvant chemoimmunotherapy and/or radiation (all p < 0.05) (**Table 1A**). Among cN2 patients, those in the ypN+ subgroup were more likely to be diagnosed with a lower clinical T stage, experience a longer wait time until initiation of neoadjuvant chemoimmunotherapy, have positive surgical margins and higher post-therapy pathologic T staging, and receive adjuvant chemoimmunotherapy and/or radiation (all p < 0.05) (**Table 1B**).

### Survival Relative to Clinical and Pathologic Nodal Status

**Figure 3:**
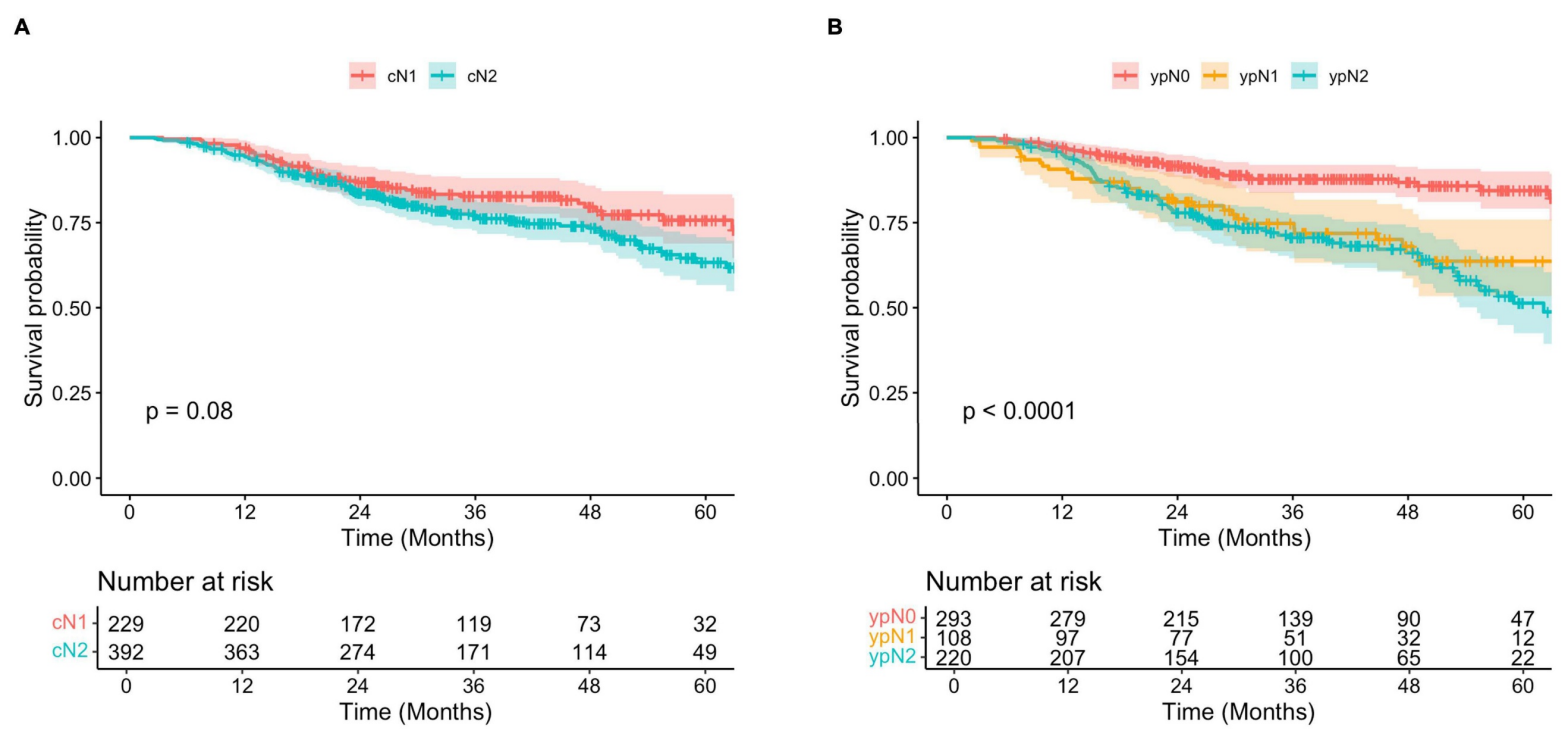
** A)** Overall survival of patients diagnosed with clinical N1 (red) versus N2 (teal) disease over 60 months. **B)** Overall survival of patients with post-therapy pathologic N0 (red), N1 (yellow), and N2 (teal) disease over 60 months.

Overall, Kaplan-Meier analysis showed that while clinically staged cN2 patients may have worse long-term survival compared to those diagnosed with cN1 disease, this difference was not statistically significant following neoadjuvant chemoimmunotherapy and surgical resection (**Figure 3A**). Five-year OS estimates were 76% (95% CI, 69-83%) and 63% (95% CI, 57-71%) for cN1 and cN2 patients, respectively (p = 0.06). In contrast, survival was significantly different when patients were stratified according to pathologic nodal status after neoadjuvant treatment, with higher staging correlating with worse OS (p < 0.001) (**Figure 3B**). Five-year OS estimates for patients with ypN0, ypN1, and ypN2 disease were 84% (95% CI, 79-90%), 64% (95% CI, 53-76%), and 51% (95% CI, 43-62%).

**Figure 4:**
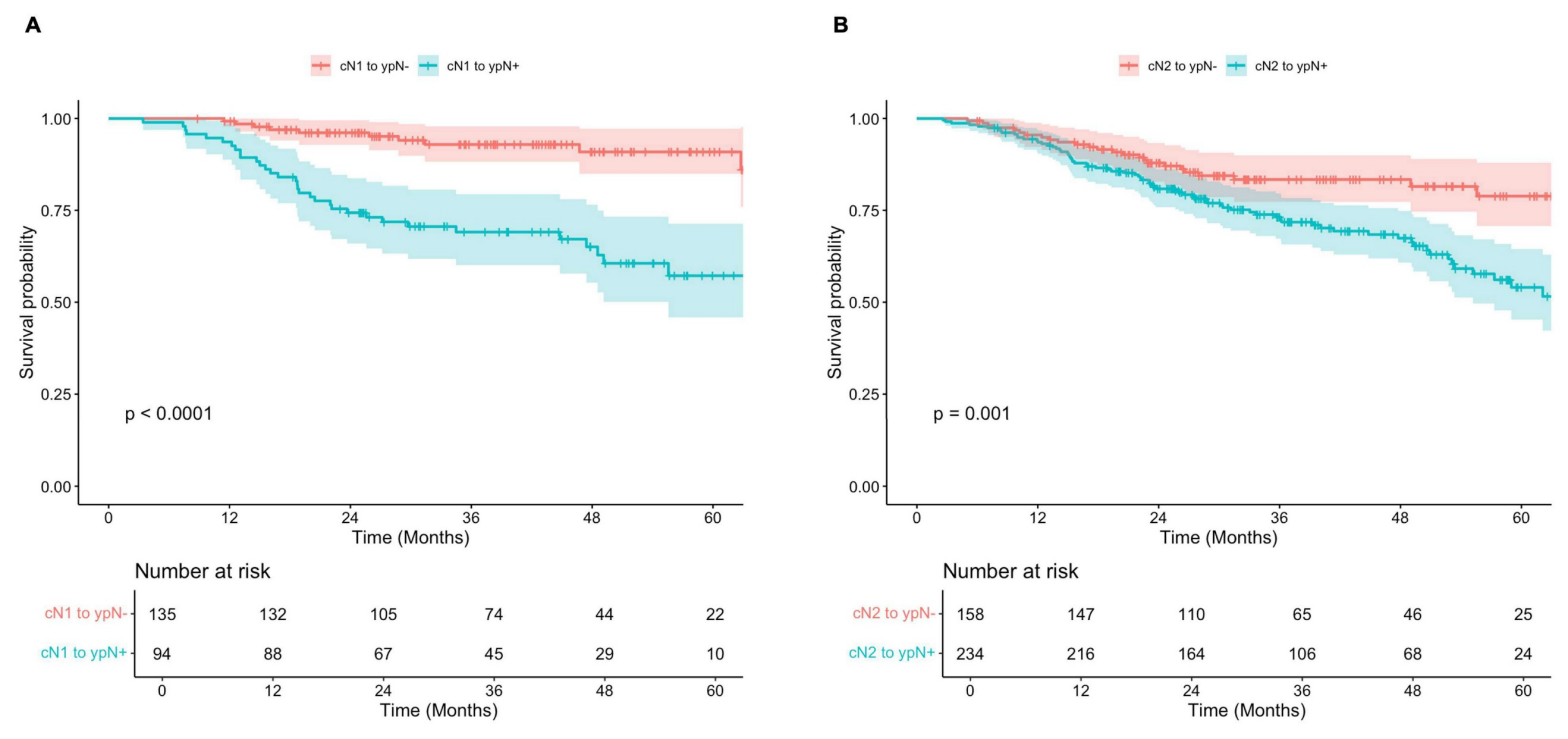
** A)** Overall survival of patients diagnosed with clinical N1 disease and negative (red) versus positive (teal) lymph nodes post-therapy.** B)** Overall survival of patients diagnosed with clinical N2 disease and negative (red) versus positive (teal) lymph nodes post-therapy.

Subgroup analyses demonstrated that for both cN1 and cN2 patients, survival was significantly improved in those who were down-staged to ypN0 (ypN-) compared to those with persistently positive nodes post-therapy (ypN+) (**Figure 4**). Among those initially diagnosed with cN1 disease, 5-year OS was 91% (95% CI, 85-97%) in the ypN- subgroup, compared to 57% (95% 46-71%) in the ypN+ subgroup (p < 0.001). Similarly, for patients initially staged as cN2, 5-year OS was 79% (95% CI, 71-88%) and 54% (95% CI, 45-64%) for those with ypN- and ypN+ disease post-therapy (p = 0.001). Of note, survival was significantly improved in patients with cN1 disease who were down-staged to ypN0 (ypN-) when compared to both ypN1 (p = 0.001) and ypN2 (p < 0.001), while no significant difference was observed between those staged at ypN1 and ypN2 post-therapy (p = 0.138). For patients initially staged as cN2, survival was significantly improved among those who were down-staged to ypN0 (ypN-) compared to those with persistent ypN2 disease (p = 0.003), but not significantly different from those who were down-staged to ypN1 (p = 0.181) (**Supplementary Figure 1**).

### Lymph Node Downstaging Predicts Survival after Neoadjuvant Chemoimmunotherapy

When taking pre- and post-therapy nodal status into consideration, univariate Cox proportional hazards analysis indicated that cN1 to ypN+ (HR, 4.35; 95% CI, 2.25-8.40; p < 0.001) and cN2 to ypN+ (HR, 4.02; 95% CI, 2.19-7.39; p < 0.001) staging were predictors of worse OS compared to cN1 to ypN- (**Table 2**). There was no significant difference in survival between cN1 or cN2 patients who were ultimately down-staged to ypN- (HR, 1.92; 95% CI, 0.97-3.82; p = 0.063). Other significant predictors of worse OS included male sex, facility type, clinical stage T4, shorter time between diagnosis and surgical resection, sublobar resection and pneumonectomy, positive surgical margin, examination of < 10 regional nodes, and post-therapy pathologic T stage ≥ T2 (all p < 0.05) (**Table 2**). After adjusting for facility type, surgical approach, surgical margin status, number of regional nodes examined, post-therapy pathologic T stage, and administration of adjuvant chemoimmunotherapy, cN1 to ypN+ (HR, 2.56; 95% CI, 1.26-5.18; p = 0.009) and cN2 to ypN+ (HR, 3.09; 95% CI, 1.60-5.99; p = 0.001) remained significant predictors of worse OS compared to cN1 to ypN- staging. Notably, cN2 to ypN- became a significant predictor of worse survival (HR, 2.80; 95% CI, 1.17-6.75; p = 0.021) after adjusting for the above factors (**Table 3**).

**Figure 5:**
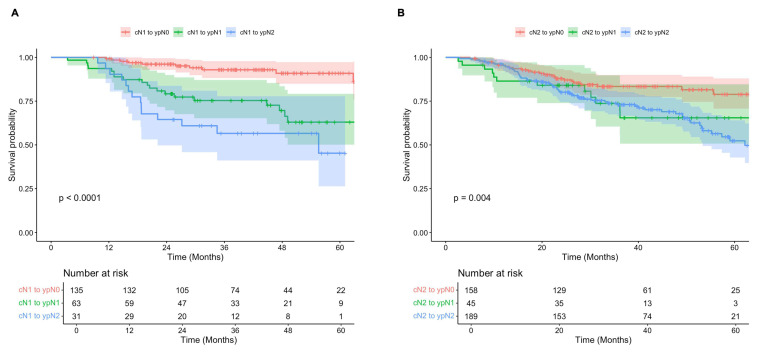
** A)** Overall survival of patients with negative lymph nodes post-therapy who received adjuvant chemoimmunotherapy (teal) versus those who did not (red). **B)** Overall survival of patients with positive lymph nodes post-therapy who received adjuvant chemoimmunotherapy (teal) versus those who did not (red).

Additional Kaplan-Meier analyses examining the effect of adjuvant chemoimmunotherapy on survival showed no difference in 5-year OS between patients who did and did not receive adjuvant chemoimmunotherapy after experiencing complete nodal response (82% versus 86%, p = 0.260) (**Figure 5A**). For patients with persistently positive nodes (ypN+), 5-year OS was increased in those who underwent adjuvant chemoimmunotherapy, but the difference not statistically significant (60% versus 49%, p = 0.091) (**Figure 5B**).

## Discussion

This study was conducted to evaluate the prognostic value of nodal response following the administration of neoadjuvant chemoimmunotherapy. Results indicated that for patients with resectable NSCLC, post-therapy nodal status was a more significant predictor of long-term survival than clinical N stage. These findings reflect the true survival impact of lymph node down-staging for contemporary patients receiving neoadjuvant chemoimmunotherapy. Within this cohort, 59% of those who were initially staged as cN1 and 40% of those staged as cN2 experienced complete nodal response or downstaging to ypN0 post-therapy. For patients diagnosed as cN1, complete nodal response conferred a significant survival benefit with a 5-year OS of 91%, compared to 57% among those with positive nodes post-therapy (p < 0.001). Similarly, survival was improved for cN2 patients with negative nodes post-therapy in comparison to those with persistent nodal disease, as demonstrated by a 5-year OS of 79% versus 54% (p = 0.001). Nodal downstaging remained a significant predictor of OS when adjusting for treatment facility type, surgical approach, surgical margin status, number of regional lymph nodes removed and examined, post-therapy pathologic T stage, and administration of adjuvant chemoimmunotherapy. Finally, among patients experiencing complete nodal response, survival was not significantly different between those who received adjuvant systemic therapy and those who did not. These findings bring into question the benefit of continuing ICI therapy after surgical resection among patients who experience nodal downstaging to ypN0 following neoadjuvant chemoimmunotherapy.

In recent years, the introduction of ICIs and other targeted agents has revolutionized the management of NSCLC, establishing immunotherapy as a principal constituent of first-line therapy 7,9. The combination of immunotherapy with chemotherapy as a neoadjuvant treatment strategy has led to unprecedented rates of complete or major pathologic response for patients with NSCLC, correlating with improvements in long-term survival. For instance, the CheckMate 816 trial showed that patients who received neoadjuvant nivolumab plus chemotherapy experienced longer event-free survival (31.6 months vs. 20.8 months) and significantly higher rates of pathologic complete response (24.0% vs. 2.2%) compared to those who received chemotherapy alone 10. Similar findings were reported in the KEYNOTE-671 trial for pembrolizumab (47.2 months vs. 18.3 months for median event-free survival and 18% vs. 4% for rate of pathologic complete response) 11; the AEGEAN trial for durvalumab (not reached vs. 25.9 months and 17.2% vs. 4.3%) 12; and the Neotorch trial for toripalimab (not reached vs. 15.1 months and 24.8% vs. 1.0%) 13. Furthermore, a study by Martins *et al.* using retrospective data from the NCDB found that patients with NSCLC who received neoadjuvant chemoimmunotherapy had significantly improved survival over those who received chemoimmunotherapy only after resection 22. In our study, 17% of the total cohort treated with neoadjuvant chemoimmunotherapy achieved pathologic complete response (ypT0N0), which is consistent with the rates in previous reports.

The efficacy of contemporary neoadjuvant treatment regimens involving ICIs plus chemotherapy, in addition to the high rate of nodal downstaging and its prognostic significance, may indicate a need to reevaluate the role of initial lymph node staging in patient selection for surgery. Specifically, routine administration of neoadjuvant chemoimmunotherapy for node-positive NSCLC could broaden the population of patients eligible for surgical resection as post-therapy nodal status is more prognostically relevant than clinical N stage. For instance, a recent retrospective analysis of patients with borderline resectable NSCLC, including those with T4 tumors and N3 disease, demonstrated high rates of complete pathologic response (29%) and surgical resectability (75%) after neoadjuvant ICI with chemotherapy 23.

Additionally, for patients exhibiting complete or major pathologic response within the primary tumor or lymph nodes, further systemic therapy may be better guided by the results of post-therapy rather than preoperative staging. Potentially, patients achieving ypN0 after neoadjuvant chemoimmunotherapy may be treated similarly to those who were initially staged as cN0, without further need for systemic therapy, though future comparative studies are necessary to determine the validity of this practice. Of note, while adjuvant therapy was optional in the CheckMate 816 trial 10, the majority of studies investigating neoadjuvant chemoimmunotherapy for NSCLC involve the use of immunotherapy both before and after surgery 9,11-13.

Results from our study are congruent with published data showing a significant and independent correlation between complete nodal response and improved survival in patients who have received neoadjuvant chemotherapy for NSCLC 19-21. For instance, Corsini *et al.* found that among patients exhibiting major pathologic response within the primary tumor, those with negative nodes post-therapy had prolonged disease-free survival compared to those with positive nodes 21. A recent retrospective study examining neoadjuvant chemoimmunotherapy for resectable NSCLC by Ma *et al.* also reported similar findings in a Chinese population, with significantly worse disease-free and overall survival associated with ypN+ status compared to ypN0 24. Interestingly, they observed no difference in outcomes between patients who were initially diagnosed as cN0 and remained node-negative versus those with positive nodes pretreatment who were subsequently down-staged to ypN0. It is also worth noting that among ypN0 patients in this cohort, those exhibiting major pathologic response in the primary tumor had significantly improved disease-free survival, as well as a trend towards better OS, compared to those who did not 24. In our analyses, both post-therapy pathologic T and N stages were independent predictors of long-term survival.

In 2014, Hellman *et al.* proposed the use of major pathologic response as a surrogate endpoint in place of OS for trials where long follow-up times could delay clinical progress 25. While major pathologic response is now a widely accepted metric for oncologic outcomes, the importance of residual tumor in nodal tissue may be independently significant, even among patients experiencing substantial downstaging of the primary tumor 26. It is appreciated that nodal or distant metastases could represent distinct clonal subpopulations that have undergone genomic evolution, harboring oncogenic mechanisms of resistance unique to the primary tumor 27,28. As a result, the significance of a major pathologic response may be undercut by the persistence of particularly resistant or aggressive populations of micrometastatic cancer cells present in the lymph nodes.

Our study reinforces lymph node downstaging as a valuable biomarker for both treatment efficacy and postsurgical survival in contemporary patients treated with neoadjuvant chemoimmunotherapy for NSCLC. Identification of specific factors associated with successful nodal downstaging will be necessary to improve patient selection criteria for both neoadjuvant chemoimmunotherapy and operative intervention, particularly for those with borderline resectable tumors. As expected, higher post-therapy T stage was independently associated with worse OS for both ypN- and ypN+ patients, indicating that pathologic response of the primary tumor must also be taken into consideration. Focused evaluation of patients experiencing pathologic complete response could further elucidate the prognostic significance of tumor and nodal downstaging, as well as the role of postoperative systemic therapies in this population. Future research integrating molecular and immunologic biomarkers may also enhance our understanding of treatment efficacy and facilitate more personalized therapeutic strategies for NSCLC.

*Limitations*. Our study carries several inherent limitations. First, the potential for unmeasured confounding exists due to its retrospective nature. Second, we were unable to incorporate *EGFR* and *ALK* mutation or PD-1 status into our analysis given the dearth or absence of recorded values for these variables in the NCDB. Third, while the accuracy of clinical staging in the NCDB is consistently high, especially for patients with positive nodes, we were unable to assess the burden or spread of lymph node disease and whether bulky or multi-station disease was present. Fourth, we unable to determine and account for patient attrition and chemoimmunotherapy regimen completion, type of immunotherapy administered, nor the number of cycles of systemic therapy patients received. Fifth, the NCDB does not provide information regarding cause of death or tumor recurrence, precluding the calculation of event-free, disease-free, or progression-free survival. Finally, we were unable to perform adequately powered subgroup analyses to evaluate the impact of adjuvant therapy on the long-term outcomes of specific high-risk populations within the cohort of patients experiencing complete nodal response, such as those with advanced post-therapy tumor staging (ypT3 and ypT4), due to small sample size.

*Conclusion*. For patients receiving neoadjuvant chemoimmunotherapy for resectable locoregionally advanced NSCLC, lymph node downstaging serves as a significant independent predictor of long-term survival in conjunction with post-therapy staging of the primary tumor. In patients experiencing complete nodal response, administration of postoperative chemoimmunotherapy was not associated with a significant difference in survival. These findings highlight the importance of nodal response as a prognostic indicator when stratifying patients for surgical resection and adjuvant therapies.

## Supplementary Material

Supplementary figure.

## Figures and Tables

**Table 1A:** Clinical N1 Patient Demographic and Clinical Characteristics

Variable	Total (N = 229)	ypN- (N = 135)	ypN+ (N = 94)	p-value
Age at diagnosis, years	65 (59, 70)	66 (60, 70)	63 (56, 70)	0.046
Sex, female	108 (47%)	75 (56%)	33 (35%)	0.002*
Race				0.480
White	203 (89%)	122 (90%)	81 (86%)	
Black	12 (5.2%)	7 (5.2%)	5 (5.3%)	
Other	14 (6.1%)	6 (4.4%)	8 (8.5%)	
Facility type				0.134
Community cancer program	10 (4.5%)	3 (2.3%)	7 (7.6%)	
Comprehensive community cancer program	48 (21%)	33 (25%)	15 (16%)	
Academic or research program	135 (60%)	77 (58%)	58 (63%)	
Integrated network cancer program	31 (14%)	19 (14%)	12 (13%)	
Insurance status				0.244
Private	110 (49%)	61 (46%)	49 (53%)	
Government	114 (51%)	71 (54%)	43 (46%)	
Uninsured	1 (0.4%)	0 (0%)	1 (1.1%)	
Charlson-Deyo comorbidity index score				0.078
0	147 (64%)	87 (64%)	60 (64%)	
1	57 (25%)	30 (22%)	27 (29%)	
2	12 (5.2%)	11 (8.1%)	1 (1.1%)	
≥3	13 (5.7%)	7 (5.2%)	6 (6.4%)	
Histology				0.458
Adenocarcinoma	113 (49%)	63 (47%)	50 (53%)	
Squamous cell carcinoma	84 (37%)	54 (40%)	30 (32%)	
Other	32 (14%)	18 (13%)	14 (15%)	
Tumor size, cm	4.4 (2.6, 6.4)	4.3 (2.4, 6.3)	4.5 (2.9, 6.6)	0.347
Clinical T stage				0.536
cT1	53 (24%)	33 (25%)	20 (22%)	
cT2	51 (23%)	28 (21%)	23 (25%)	
cT3	78 (35%)	50 (38%)	28 (31%)	
cT4	42 (19%)	22 (17%)	20 (22%)	
Days from diagnosis to start of systemic therapy	45 (32, 63)	48 (34, 62)	45 (30, 63)	0.501
Days from diagnosis to start of immunotherapy	55 (35, 106)	53 (35, 95)	57 (32, 132)	0.764
Days from diagnosis to surgical resection	137 (100, 181)	143 (111, 188)	120 (80, 177)	0.003*
Operation				0.002*
Lobectomy or bilobectomy	200 (87%)	126 (93%)	74 (79%)	
Wedge or segmental resection	13 (5.7%)	6 (4.4%)	7 (7.4%)	
Pneumonectomy	16 (7.0%)	3 (2.2%)	13 (14%)	
Surgical approach				0.272
Robotic-assisted thoracoscopic	59 (30%)	41 (34%)	18 (23%)	
Video-assisted thoracoscopic	41 (21%)	25 (20%)	16 (21%)	
Open thoracotomy	99 (50%)	56 (46%)	43 (56%)	
Unknown	30 (13%)	13 (10%)	17 (18%)	
Postoperative length of stay, days	4 (2, 5)	4 (2, 5)	4 (2, 6)	0.641
30-day postoperative mortality	0 (0%)	0 (0%)	0(0%)	1.000
90-day postoperative mortality	3 (1.3%)	1 (0.7%)	2 (2.1%)	0.747
Surgical margins				< 0.001*
Negative	213 (95%)	133 (99%)	80 (88%)	
Positive	12 (5.3%)	1 (0.7%)	11 (12%)	
Microscopic residual tumor	7 (3.1%)	0 (0%)	7 (7.7%)	
Macroscopic residual tumor	5 (2.2%)	1 (0.7%)	4 (4.4%)	
Regional nodes examined	15 (10, 23)	13 (9, 22)	18 (11, 24)	0.037*
Regional nodes positive	1 (0, 3)	0 (0, 0)	2 (1, 4)	<0.001*
Post-therapy pathologic T stage				<0.001*
ypT0	54 (24%)	48 (36%)	6 (6.5%)	
ypT1	74 (33%)	49 (36%)	25 (27%)	
ypT2	46 (20%)	16 (12%)	30 (33%)	
ypT3	38 (17%)	20 (15%)	18 (20%)	
ypT4	15 (6.6%)	2 (1.5%)	13 (14%)	
Adjuvant chemoimmunotherapy	101 (44%)	49 (36%)	52 (55%)	0.004*
Adjuvant radiation	31 (14%)	11 (8.3%)	20 (22%)	0.004*

**Table 1B:** Clinical N2 Patient Demographic and Clinical Characteristics

Variable	Total (N = 392)	ypN- (N = 158)	ypN+ (N =234)	p-value
Age at diagnosis, years	64 (58, 71)	64 (58, 71)	65 (59, 71)	0.869
Sex, female	204 (52%)	84 (53%)	120 (51%)	0.714
Race				0.633
White	334 (85%)	136 (86%)	198 (85%)	
Black	30 (7.7%)	13 (8.2%)	17 (7.3%)	
Other	28 (7.1%)	9 (5.7%)	19 (8.1%)	
Facility type				0.414
Community cancer program	18 (4.7%)	10 (6.5%)	8 (3.5%)	
Comprehensive community cancer program	77 (20%0	27 (17%)	50 (22%)	
Academic or research program	242 (63%)	97 (63%)	145 (63%)	
Integrated network cancer program	48 (12%)	21 (14%)	27 (12%)	
Insurance status				1.000
Private	167 (43%)	67 (44%)	100 (43%)	
Government	216 (56%)	86 (56%)	130 (56%)	
Uninsured	2 (0.5%)	1 (0.6%)	1 (0.4%)	
Charlson-Deyo comorbidity index score				0.514
0	264 (67%)	106 (67%)	158 (68%)	
1	83 (21%)	34 (22%)	49 (21%)	
2	33 (8.4%)	11 (7.0%)	22 (9.4%)	
≥3	12 (3.1%)	7 (4.4%)	5 (2.1%)	
Histology				0.990
Adenocarcinoma	220 (56%)	88 (56%)	132 (56%)	
Squamous cell carcinoma	103 (26%)	42 (27%)	61 (26%)	
Other	69 (18%)	28 (18%)	41 (18%)	
Tumor size, cm	3.5 (2.2, 5.2)	3.5 (2.2, 6.2)	3.5 (2.2, 4.9)	0.213
Clinical T stage				0.043*
cT1	130 (34%)	47 (30%)	83 (36%)	
cT2	134 (35%)	47 (30%)	87 (37%)	
cT3	74 (19%)	33 (21%)	41 (18%)	
cT4	50 (13%)	28 (18%)	22 (9.4%)	
Days from diagnosis to start of systemic therapy	49 (33, 67)	51 (32, 72)	47 (34, 65)	0.372
Days from diagnosis to start of immunotherapy	69 (40, 198)	60 (37, 106)	84 (43, 219)	< 0.001*
Days from diagnosis to surgical resection	149 (119, 185)	161 (135, 204)	140 (111, 169)	< 0.001*
Operation				0.145
Lobectomy or bilobectomy	335 (86%)	140 (89%)	195 (83%)	
Wedge or segmental resection	23 (5.9%)	9 (5.7%)	14 (6.0%)	
Pneumonectomy	33 (8.4%)	8 (5.1%)	25 (11%)	
Surgical approach				0.299
Robotic-assisted thoracoscopic	106 (31%)	42 (30%)	64 (33%)	
Video-assisted thoracoscopic	74 (22%)	37 (26%)	37 (19%)	
Open thoracotomy	157 (47%)	63 (44%)	94 (48%)	
Unknown	55	16	39	
Postoperative length of stay, days	4 (3, 6)	4 (3, 6)	4 (3, 6)	0.407
30-day postoperative mortality	7 (1.8%)	3 (1.9%)	4 (1.7%)	0.124
90-day postoperative mortality	12 (3.1%)	6 (3.8%)	6 (2.6%)	0.076
Surgical margins				0.002*
Negative	350 (91%)	151 (97%)	199 (88%)	
Positive	33 (8.6%)	5 (3.2%)	28 (12%)	
Microscopic residual tumor	13 (3.4%)	1 (0.6%)	12 (5.3%)	
Macroscopic residual tumor	19 (5.0%)	3 (1.9%)	16 (7.0%)	
Regional nodes examined	15 (9, 23)	13 (7, 20)	17 (10, 24)	< 0.001*
Regional nodes positive	2 (0, 5)	0 (0, 1)	3 (2, 6)	< 0.001*
Post-therapy pathologic T stage				< 0.001*
ypT0	75 (19%)	60 (39%)	15 (6.5%)	
ypT1	146 (38%)	61 (39%)	85 (37%)	
ypT2	91 (24%)	17 (11%)	74 (32%)	
ypT3	44 (11%)	7 (4.5%)	37 (16%)	
ypT4	31 (8.0%)	10 (6.5%)	21 (9.1%)	
Adjuvant chemoimmunotherapy	204 (52%)	63 (40%)	141 (60%)	< 0.001*
Adjuvant radiation	136 (35%)	19 (12%)	117 (50%)	< 0.001*

**Table 2:** Univariate Cox Proportional Hazards Analysis for Significant Predictors of Long-Term Survival

Variable	Hazard Ratio	p-value
Nodal status		
cN1 to ypN-	Ref	Ref
cN1 to ypN+	4.35 (2.25-8.40)	< 0.001*
cN2 to ypN-	1.92 (0.97-3.83)	0.063
cN2 to ypN+	4.02 (2.19-7.39)	< 0.001*
Age	1.01 (0.99-1.03)	0.198
Female sex	0.66 (0.47-0.91)	0.011*
Race		
White	Ref	Ref
Black	1.27 (0.69-2.36)	0.444
Other	1.01 (0.51-1.98)	0.981
Charlson-Deyo comorbidity index		
0	Ref	Ref
1	1.01 (0.68-1.51)	0.944
2	1.20 (0.64-2.23)	0.576
≥3	1.58 (0.77-3.26)	0.214
Facility type		
Community cancer program	Ref	Ref
Comprehensive community cancer program	0.60 (0.30-1.23)	0.164
Academic or research program	0.44 (0.23-0.86)	0.016*
Integrated network cancer program	0.43 (0.19-0.96)	0.039*
Histology		
Adenocarcinoma	Ref	Ref
Squamous cell carcinoma	1.19 (0.82-1.72)	0.356
Other	1.18 (0.75-1.86)	0.468
Clinical T stage		
cT1	Ref	Ref
cT2	1.63 (1.04-2.56)	0.032*
cT3	1.24 (0.76-2.03)	0.391
cT4	2.25 (1.37-3.70)	0.001*
Months from diagnosis to start of systemic therapy	0.93 (0.81-1.07)	0.307
Months from diagnosis to start of immunotherapy	1.02 (0.98-1.05)	0.304
Months from diagnosis to surgical resection	0.91 (0.83-0.98)	0.019*
Operation		
Lobectomy or bilobectomy	Ref	Ref
Wedge or segmental resection	2.31 (1.30-4.12)	0.004*
Pneumonectomy	3.12 (2.04-4.76)	< 0.001*
Positive surgical margin	3.07 (1.96-4.82)	< 0.001*
Regional nodes examined		
< 10	Ref	Ref
≥ 10	0.64 (0.45-0.93)	0.018*
Post-therapy pathologic T stage		
ypT0	Ref	Ref
ypT1	1.59 (0.82-3.08)	0.169
ypT2	3.49 (1.84-6.60)	< 0.001*
ypT3	3.70 (1.87-7.33)	< 0.001*
ypT4	8.36 (4.27-16.4)	< 0.001*
Adjuvant chemoimmunotherapy	1.04 (0.75-1.43)	0.821
Adjuvant radiation	1.29 (0.92-1.82)	0.141

**Table 3:** Multivariate Cox Proportional Hazards Analysis for Significant Predictors of Long-Term Survival

Variable	Hazard Ratio	p-value
Nodal status		
cN1 to ypN-	Ref	Ref
cN1 to ypN+	4.04 (1.68-9.74)	0.002*
cN2 to ypN-	2.80 (1.17-6.74)	0.021*
cN2 to ypN+	4.49 (1.99-10.1)	< 0.001*
Facility type		
Community cancer program	Ref	Ref
Comprehensive community cancer program	0.60 (1.18-2.05)	0.413
Academic or research program	0.49 (0.15-1.59)	0.233
Integrated network cancer program	0.44 (0.12-1.65)	0.222
Surgical approach		
Minimally invasive	Ref	Ref
Open thoracotomy	1.52 (1.01-2.31)	0.047*
Positive surgical margin	1.81 (1.04-3.15)	0.035*
Regional lymph nodes examined >10	0.80 (0.54-1.20)	0.282
Post-therapy pathologic T stage		
ypT0	Ref	Ref
ypT1	1.64 (0.70-3.88)	0.255
ypT2	2.92 (1.22-7.01)	0.016*
ypT3	3.87 (1.56-9.58)	0.004*
ypT4	4.74 (1.83-12.3)	0.001*
Adjuvant chemoimmunotherapy	0.81 (0.55-1.20)	0.287
