# Supplementary material for: The Prognostic Value of Lymph Node Downstaging Following Neoadjuvant Chemoimmunotherapy for Non-Small Cell Lung Cancer
Source: J Cancer. 2025 Oct 20;16(15):4292–301. doi: 10.7150/jca.119881 (PMC12664728; doi:10.7150/jca.119881)
Supplement: Supplementary file 1 — Supplementary figure. [file jcav16p4292s1.pdf]

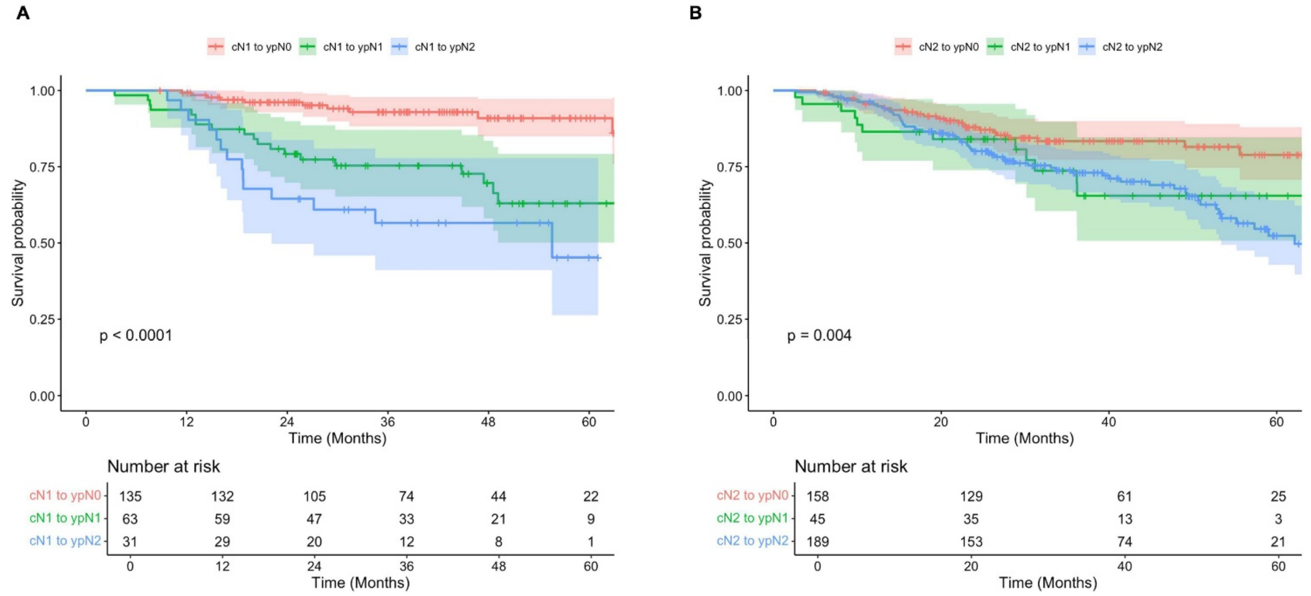

Supplementary Figure 5. A) Overall survival of patients diagnosed with clinical N1 disease and post-therapy pathologic stages ypN0 (red), ypN1 (green), and ypN2 (blue). **B)** Overall survival of patients diagnosed with clinical N2 disease and post-therapy pathologic stages ypN0 (red), ypN1 (green), and ypN2 (blue).
